# Disbalanced recruitment of crossed and uncrossed cerebello-thalamic pathways during deep brain stimulation is predictive of delayed therapy escape in essential tremor

**DOI:** 10.1016/j.nicl.2024.103576

**Published:** 2024-02-12

**Authors:** Bastian E.A. Sajonz, Marvin L. Frommer, Marco Reisert, Ganna Blazhenets, Nils Schröter, Alexander Rau, Thomas Prokop, Peter C. Reinacher, Michel Rijntjes, Horst Urbach, Philipp T. Meyer, Volker A. Coenen

**Affiliations:** a Department of Stereotactic and Functional Neurosurgery, Medical Center – University of Freiburg, Faculty of Medicine, University of Freiburg, Freiburg im Breisgau, Germany; b Division of Medical Physics, Department of Diagnostic and Interventional Radiology, University Medical Center Freiburg, Faculty of Medicine, University of Freiburg, Germany; c Department of Nuclear Medicine, Medical Center – University of Freiburg, Faculty of Medicine, University of Freiburg, Freiburg im Breisgau, Germany; d Department of Neurology and Neurophysiology, Medical Center – University of Freiburg, Faculty of Medicine, University of Freiburg, Freiburg im Breisgau, Germany; e Department of Neuroradiology, Medical Center – University of Freiburg, Faculty of Medicine, University of Freiburg, Freiburg im Breisgau, Germany; f Fraunhofer Institute for Laser Technology (ILT), Aachen, Germany; g Center for Deep Brain Stimulation, University of Freiburg, Germany

**Keywords:** Ataxia, Cerebello-thalamic projection pathways, Deep brain stimulation, Delayed therapy escape, Dentato-rubro-thalamic tract, Essential tremor, Tremor network

## Abstract

**Background:**

Thalamic deep brain stimulation (DBS) is an efficacious treatment for drug-resistant essential tremor (ET) and the dentato-rubro-thalamic tract (DRT) constitutes an important target structure. However, up to 40% of patients habituate and lose treatment efficacy over time, frequently accompanied by a stimulation-induced cerebellar syndrome. The phenomenon termed delayed therapy escape (DTE) is insufficiently understood. Our previous work showed that DTE clinically is pronounced on the non-dominant side and suggested that differential involvement of crossed versus uncrossed DRT (DRTx/DRTu) might play a role in DTE development.

**Methods:**

We retrospectively enrolled right-handed patients under bilateral thalamic DBS >12 months for ET from a cross-sectional study. They were characterized with the Fahn-Tolosa-Marin Tremor Rating Scale (FTMTRS) and Scale for the Assessment and Rating of Ataxia (SARA) scores at different timepoints. Normative fiber tractographic evaluations of crossed and uncrossed cerebellothalamic pathways and volume of activated tissue (VAT) studies together with [^18^F]Fluorodeoxyglucose positron emission tomography were applied.

**Results:**

A total of 29 patients met the inclusion criteria. Favoring DRTu over DRTx in the non-dominant VAT was associated with DTE (R^2^ = 0.4463, p < 0.01) and ataxia (R^2^ = 0.2319, p < 0.01). Moreover, increasing VAT size on the right (non-dominant) side was associated at trend level with more asymmetric glucose metabolism shifting towards the right (dominant) dentate nucleus.

**Conclusion:**

Our results suggest that a disbalanced recruitment of DRTu in the non-dominant VAT induces detrimental stimulation effects on the dominant cerebellar outflow (together with contralateral stimulation) leading to DTE and thus hampering the overall treatment efficacy.

## Highlights

• Addressing DRTx with VIM-DBS is associated with long-term efficacy.
• Coverage of DRTu by non-dominant VAT is associated with delayed therapy escape.
• Initial efficacy of DRTu seems to be misleading during intraoperative testing.

## 1 Introduction

Delayed therapy escape (DTE) after thalamic deep brain stimulation (DBS) for essential tremor (ET) affects up to 30–40 % of patients (Chiu et al., 2020, Pilitsis et al., 2008). Patients are troubled with tremor of preoperative severity (or worse) ≥12 months after treatment installation (Fasano and Helmich, 2019). DTE constitutes a complex phenomenon, difficult to deconstruct on anatomical and physiological grounds. Several factors seem to be involved amongst them electrode positioning (Anthofer et al., 2017, Favilla et al., 2012, Papavassiliou et al., 2004, Pilitsis et al., 2008). Habituation of tremor control is the primary symptom of DTE. In our experience, this is followed by loss of dexterity, gait disturbance and dysarthria which are typically not present in the initial stimulation phase. These symptoms point to a dysfunction of the cerebellum and the experienced time delay between a stimulation adjustment (increased amplitude) and the occurrence of these symptoms indicates an involvement of DBS (Sajonz et al., 2022).

There is increasing evidence that DBS for ET should address the dentato-rubro-thalamic tract (DRT) to be efficacious (Akram et al., 2018, Al-Fatly et al., 2019, Coenen et al., 2020, Coenen et al., 2017, Coenen et al., 2014, Coenen et al., 2011a, Coenen et al., 2011b, Dembek et al., 2020, Deuter et al., 2022, Middlebrooks et al., 2022, Middlebrooks et al., 2021, Nowacki et al., 2018, Schlaier et al., 2015). Most of these studies focus on the larger decussating portion of the tract (DRTx). However, there is indication from animal research, microdissection and imaging studies (Ilinsky et al., 1987, Meola et al., 2016, Petersen et al., 2018) that a smaller portion proceeds ipsilaterally from the dentate nucleus (DN) to the ventral thalamus (DRTu, uncrossed). One month postoperatively, efficacious DBS contacts in ET for the intentional tremor component were found in proximity to the DRTx, whereas electrode contacts efficacious for the postural tremor component were located in proximity to both DRTx and DRTu (Deuter et al., 2022). Tsuboi et al. (2021) showed that the relation of DRTx to DRTu within the stimulation field plays a role when treating dystonic tremor, while in ET both portions seem to be equally efficacious in the early postoperative course up to 12 months. Most studies examining efficacy of thalamic DBS focus on a 12 months follow up (FU) period only, thereby circumventing the need to report potential DTE. With respect to detrimental and long-term stimulation side effects, a differential recruitment of distinct portions of the DRT has not been reported.

In this retrospective study, we examined how differential recruitment contributes to the evolution of DTE by utilizing normative fiber tractographic evaluations of DRTx and DRTu, volume of activated tissue (VAT) studies and [^18^F]Fluorodeoxyglucose ([^18^F]FDG) positron emission tomography (PET). DTE seems to be pronounced on the non-dominant hand (Sajonz et al., 2022), hence, we hypothesized that fractional recruitment of DRTu in the non-dominant thalamus might be associated with habituation and consecutive DTE.

## 2 Methods

### 2.1 Patients and design of analysis

In this work, we analyzed prospective data of a cross-sectional study on patients with bilateral thalamic DBS >12 months for ET. The study follows the tenets of the declaration of Helsinki and was approved by the local ethics committee (623/19). Thirty-one patients provided written informed consent. Prospective data collection consisted of stimulation parameters and the clinical test battery mentioned below.

For the analyses of this publication, we defined right-handedness as an additional inclusion criterion resulting in a sample of 29 patients. Whenever available, we included retrospective clinical data from the following points: (1) preoperative (“preOP”), (2) 12 months postoperative (“12 M”), (3) before in-patient stimulation adjustment. As the in-patient adjustments resulted in significant stimulation parameter changes including the introduction of pulse width reduction and in some cases additional surgery on the DBS-system, we focussed this analysis on the situation before these changes to avoid bias. Therefore, we grouped the retrospective data acquired on admission just before in-patient stimulation adjustment (n = 16) together with the prospectively collected study data of 13 patients without prior in-patient stimulation adjustment and termed it late FU (“late”). We analyzed the stimulation ON state except, of course, for the preOP point.

If applied to the preoperative situation of all patients, the consensus criteria of Bhatia et al. (2018) identify ET in 26 patients and ETplus in three patients.

### 2.2 Clinical outcomes

#### 2.2.1 Test battery

The Fahn-Tolosa-Marin Tremor Rating Scale Severity and Performance Parts (A and B) (FTMTRS) (Fahn et al., 1993) and the Scale for the Assessment and Rating of Ataxia (SARA) (Schmitz-Hübsch et al., 2006) were used to measure tremor and ataxia. All of these examinations were conducted at our center by the following authors: all preOP examinations by TP, 12M examinations mostly by TP and some by BEAS, FU_late_ examinations by BEAS and MLF. 12 of the FTMTRS and SARA values from FU_late_ were mean values of video ratings done by BEAS and MLF in a blinded fashion as previously reported (Sajonz et al., 2022). The remaining FTMTRS and SARA values were acquired by an unblinded examiner (BEAS and MLF). For analysis, we used a modified SARA score without item 6 (nose-finger test measuring tremor) as previously described (Roque et al., 2021). Operationalizing the definition of DTE proposed by Fasano & Helmich (2019), we included a ratio in the analysis comparing ON_late_ vs. preOP indicating the extent of therapy efficacy (<1) and DTE (≥1), respectively. Patients were also asked at FU_late_, which hand was most affected in their opinion (i.e. causing more trouble).

#### 2.2.2 Stimulation parameters

Stimulation parameters were obtained from all included study points. The current draw from the battery was calculated for FU_late_ according to Zhang et al. (2020).

#### 2.2.3 Medication

If available, tremor medication was obtained for the FU_late_.

### 2.3 Imaging

#### 2.3.1 Processing of HCP-Data for DRT delineation

DRT templates were generated on data sets (n = 181) from the Human Connectome Project (HCP) repository utilizing global tractography (GT). Tractography was performed individually for each HCP-subject as previously described (Coenen et al., 2018, Hosp et al., 2019) based on the approach by Reisert et al., (2011, see also https://www.uniklinik-freiburg.de/mr-en/research-groups/diffperf/fibertools.html). The minimally preprocessed diffusion data was used (for more details see Glasser et al. (2013)), no further preprocessing was applied. As opposed to local walker-based tractography, GT aims to find a fiber configuration that delivers the best explanation for the acquired diffusion-weighted magnetic resonance imaging (DWI) data. From the two parameter sets provided by the toolbox (Reisert et al., 2011), we chose the ‘dense’ preset for our analyses. In addition, to increase reproducibility, we increased the number of fibers using the following accumulation strategy: after the cooling-down phase, the temperature was again increased to 0.1, and the state further iterated for 107 iterations. This procedure was iterated over 10 rounds and the tracts resulting from each round were accumulated to obtain one final tractogram (Schumacher et al., 2018), based on which the DRT was constructed using three regions of interest (ROIs): nucleus ruber, superior cerebellar peduncle (SCP) and precentral gyrus. The red nuclei of both sides are represented by a spherical mask with MNI coordinates (+/-6,–22,-10) and radius 6 mm. The SCP is given by a sphere with center (+/-7,-41,-26) and a radius of 4 mm. The precentral gyrus was obtained from the Desikan-Killiany atlas (Desikan et al., 2006). Based on the three ROIs, streamlines were selected to form the DRTx and DRTu. Additionally, we added an exclusion ROI (a sphere at MNI 0,0,-9 with radius 10 mm), to avoid including falsely crossing spurious streamlines at the level of pons. For the imaging plausibility of DRTx and DRTu rendition with GT see Coenen et al. (2021). To create a group template, streamline densities were computed by means of trilinear interpolation on an isotropic matrix with 1.5 mm resolution and transformed into group-space and group averages were built of the raw streamline densities, which are used below as a template to measure the proximity to the VAT in the individual patient.

#### 2.3.2 Image acquisition and VAT analysis in patients

Postoperative helical computed tomography (CT) was acquired on Somatom Definition AS scanners (Siemens Healthineers, Erlangen, Germany), with reconstruction kernel H30s, slice thickness of 1 mm, and in-plane resolution of 0.5 mm.

Lead localization using CT data was performed using an in-house MATLAB code followed by manual verification. To map the native CT space to the group space (MNI 2009c asym), we followed the method validated by Reisert et al. (2023). In this approach, tissue probability maps are predicted by a deep convolutional network based on the CT images and deformable registration is used to warp the native tissue probability maps onto the group space. VATs were obtained from stimulation parameters with Brainlab Elements (Brainlab, Munich, Germany) and exported as DICOM images. The DICOM images were imported into the NORA imaging platform (https://www.nora-imaging.org) for further analysis and visualization. Each VAT was transformed into MNI space (by the warp obtained from above, and the overlap with the DRTx and DRTu was measured by a simple summation of the mean fiber densities for all voxels activated in a patient. Similar to Tsuboi et al. (2021), we calculated a ratio of DRTx vs. DRTu covered by the VATs of each side that was submitted to further analysis. To explore how lateralized cerebellar outflow was affected by the bilateral stimulation regime of each patient, we calculated the sum of fibers originating from each DN covered by the VATs of both sides, i.e. sum of fibers from right DN = DRTu covered by VAT R + DRTx covered by VAT L and vice versa.

#### 2.3.3 [^18^F]FDG PET imaging

PET acquisitions were performed as previously described (Sajonz et al., 2022). In short, 10-min PET scans were acquired 50 min after intravenous injection of 214±9 MBq [^18^F]FDG under DBS stimulation (Stim ON). PET scans were spatially normalized to in-house templates in MNI space (Collins et al., 1994). Each scan was proportionally scaled to individual uptake in brain parenchyma. Based on the stimulation-related metabolic changes described previously (Sajonz et al., 2022), we defined thalamus and DN ROIs and split the clusters at midline for left and right. Mean normalized [^18^F]FDG uptake in thalamus and DN ROI was calculated for each of the participants. Intra-individual asymmetry in normalized [^18^F]FDG uptake for each ROI was assessed by an asymmetry index (AI; right minus left anatomical side divided by their mean). All processing steps were implemented with an in-house MATLAB pipeline (The MathWorks, Natick, Massachusetts, US).

### 2.4 Statistical analysis

Statistical analysis was performed with GraphPad Prism 9.5.0 (GraphPad Software, San Diego, CA, USA) and R 4.3.0 (https://www.R-project.org). Linear regression analyses were applied to determine the association between the ratio DRTx/DRTu covered by the right VAT at FU_late_ and (1) the extent of DTE (or therapy efficacy) represented by the ratio FTMTRS ON_late_/preOP as well as (2) the modified SARA score without tremor item 6. To estimate the effects of age, sex, disease duration, time elapsed since DBS implantation, and tremor medication (yes vs. no), additional exploratory multiple linear regression models were calculated. Moreover, Pearson’s correlation analyses were conducted to explore (1) potential associations at FU_late_ and FU_12M_ between measures of tremor, therapy escape, ataxia, DRT coverage by VATs of both sides, cerebellar lateralization, VAT location in MNI-space and stimulation intensity as well as (2) associations between the asymmetry of glucose metabolism and ratio DRTx/DRTu or VAT sizes. Additionally, influence of age, sex, disease duration and time elapsed since DBS implantation on the latter associations was tested in multiple linear regression analyses.

## 3 Results

Demographic and clinical characteristics and their availability are provided in Table 1. PET imaging was available in 12 patients as reported before (Sajonz et al., 2022) at FU_late_. Four patients had received their DBS implantation under a former surgical team at our center and another three patients at different center.

**Table 1:** Demographic and basic clinical data.

	*N*
Sex (Male:Female)	17:12
More affected Hand_late_ (Right:Left)	8:21
Tremor Medication_late_ (yes:no)	9:20

	*mean* ± *SD (range)*
Propranolol_late_ (mg, n = 4)^2^	90±50 (40–160)
Primidone_late_ (mg, n = 3)^2^	437.5±187.5 (250–625)
Gapapentin_late_ (mg, n = 1)	1600
Topiramat_late_ (mg, n = 1)	50
Clonazepam_late_ (mg, n = 1)	1.5
Age_late_ (years)	71±8 (51–84)
Disease duration_late_ (years)	31±18 (6–67)
Time since DBS implantation_late_ (years)	5.6±3.9 (1.1–16.5)
FTMTRS_preOP_ (n = 14)	40±11 (31–67)
FTMTRS ON_12M_ (n = 12)	16±14 (0–38)
FTMTRS ON_late_ (n = 29)	28±14 (4–67)
ratio FTMTRS ON_late_/preOP (n = 14)	0.72±0.42 (0.12–1.40)
SARA without item 6 ON_late_ (n = 28)^1^	8±4 (1–16)
Current draw from battery_late_ Left (µA)	63±60 (9–243)
Current draw from battery_late_ Right (µA)	54±59 (8–304)

1 Missing ON_late_ SARA Score in one patient.

2 one patient took propranolol and primidone.

**Fig. 1:**
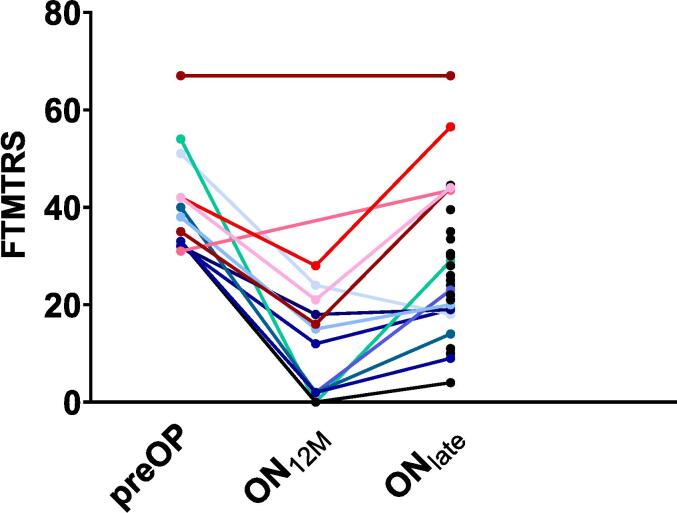
Fahn-Tolosa-Marin Tremor Rating Scale (Part A + B, FTMTRS) values over time: preoperative (preOP) in n = 14 patients, Stim ON 12 months postoperatively (ON_12M_) in n = 12 patients, Stim ON at late Follow Up (ON_late_) in n = 29 patients. Therapy escapers (n = 5) are depicted in warm colors, non-escapers in cold colors, and non-assignable patients in black. Note that time intervals between ON_12M_ and ON_late_ differ in each patient and are therefore not to scale. Medical chart review suggests that both patients with available preOP values but missing ON_12M_ values also had tremor reduction at ON_12M_.

FTMTRS values over time showed a sustained (12 months) benefit in all patients with available preOP and FU_12M_ data (Fig. 1).

**Fig. 2:**
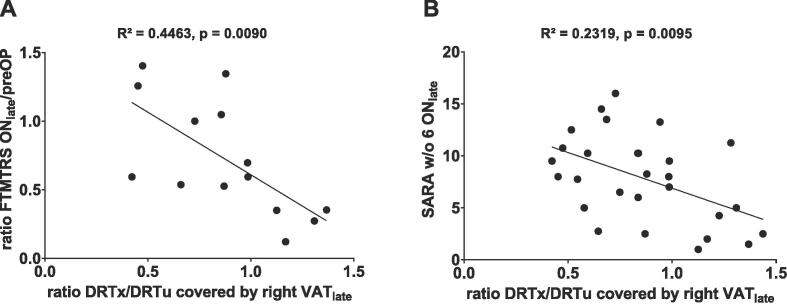
(A) Linear regression of the ratio DRTx/DRTu covered by the right VAT_late_ and the ratio of FTMTRS ON_late_/preOP (available in n = 14 patients) showing a significant negative correlation. (B) Linear regression of the ratio DRTx/DRTu covered by the right VAT_late_ and the modified SARA score without tremor item 6 ON_late_ (available in n = 28 patients) showing a significant negative correlation. Nota bene: Two patients have the same combination of values resulting in overlapping dots in graph B (x = 0.84; y = 10.25). Abbreviations: DRT = dentatorubrothalamic tract (u = non-decussating, x  = decussating), FTMTRS = Fahn-Tolosa-Marin Tremor Rating Scale (Part A + B), ON = Stim ON, preOP = preoperative, VAT = volume of activated tissue.

**Fig. 3:**
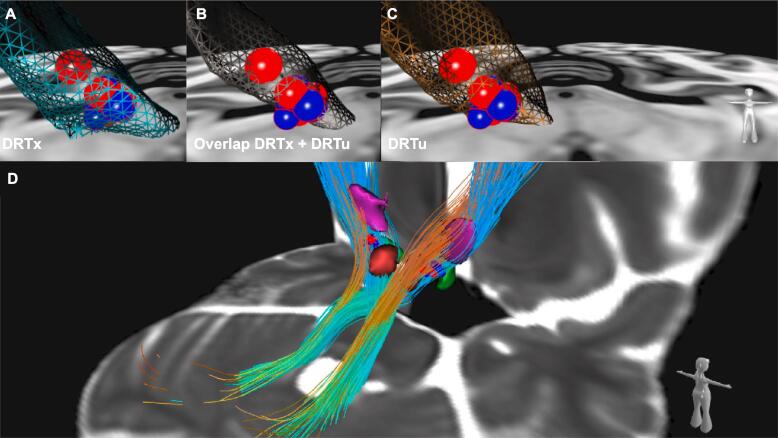
(A-C) Frontal view on right VATs from follow up_late_ of therapy escapers (red spheres) and non-escapers (blue spheres) and their overlap with the following portions of the DRT rendered as a mesh structure: (A) DRTx in light blue; (B) the overlap of DRTx and DRTu fibers in dark gray; (C) DRTu in bronze. The spatial relation is outlined best in (B): VATs of escapers tend to cover more of the overlapping region of DRTx and DRTu fibers, while VATs of non-escapers are more jutting out anteriorly, thus addressing primarily the DRTx; (D) Posterolateral view from the right giving an general overview on target regions of both sides including adjacent nuclei (derived from (Ewert et al., 2018)). DRTx and DRTu rendered as streamlines, ventral intermediate nucleus of thalamus in purple, nucleus ruber in red and subthalamic nucleus in green. Abbreviations: DRT = dentatorubrothalamic tract, u = non-decussating, x  = decussating, VAT = volume of activated tissue. (For interpretation of the references to color in this figure legend, the reader is referred to the web version of this article.)

**Fig. 4:**
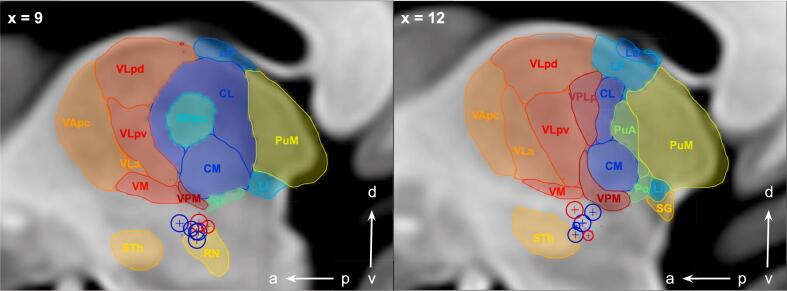
Saggital view of right-sided active electrode contact positions of therapy escapers (red spheres) and non-escapers (blue spheres) at follow up_late_ in relation to thalamic subnuclei parcellated according to the Morel stereotactic atlas (Krauth et al., 2010), where VIM location corresponds to Vlpv, Vla and Vm. Sphere diameter is set to a standard of 2 mm. Differences in diameter occur because of partial volume effects with more distant contact depiction as dots. Abbreviations: CM, Centre médian nucleus; CL, central lateral nucleus; LD, lateral dorsal nucleus; Li, limitans nucleus; LP, lateral posterior nucleus; MDpc, mediodorsal nucleus, parvocellular part; Pf, parafascicular nucleus; Po, posterior nucleus; PuA, anterior pulvinar; PuM, medial pulvinar; RN, red nucleus; SG, suprageniculate nucleus; STh, subthalamic nucleus; VApc, ventral anterior nucleus, parvocellular part; VLa, ventral lateral anterior nucleus; VLpd, ventral lateral posterior nucleus, dorsal part; VLpv, ventral lateral posterior nucleus, ventral part; VM, ventral medial nucleus; VPM, ventral posterior medial nucleus; v, ventral; d, dorsal; a, anterior; p, posterior. (For interpretation of the references to color in this figure legend, the reader is referred to the web version of this article.)

A higher ratio DRTx/DRTu covered by the right VAT_late_ was significantly associated with sustained therapeutic efficacy over time while patients with a lower ratio tended to have less therapy efficacy or even DTE (Fig. 2A and Fig. 3). Similarly, a higher ratio DRTx/DRTu was associated with a lower modified SARA score indicating less ataxia (Fig. 2B). Upon using the complete instead of the modified SARA score, the association remained significant (R^2^ = 0.2182, p = 0.0122). Multiple linear regression analyses did not reveal an influence of age, sex, disease duration, time elapsed since DBS implantation, or tremor medication on these associations. Exploratory correlational analyses (including VATs, their MNI coordinates and stimulation intensities of both sides) at FU_late_ and FU_12M_ are provided in Supplementary Sections S1 and S2. The distribution of right-sided active electrode contact positions of therapy escapers and non-escapers with regard to thalamic subnuclei is depicted in Fig. 4.

### 3.1 Exploratory evaluation of regional cerebral metabolism

**Fig. 5:**
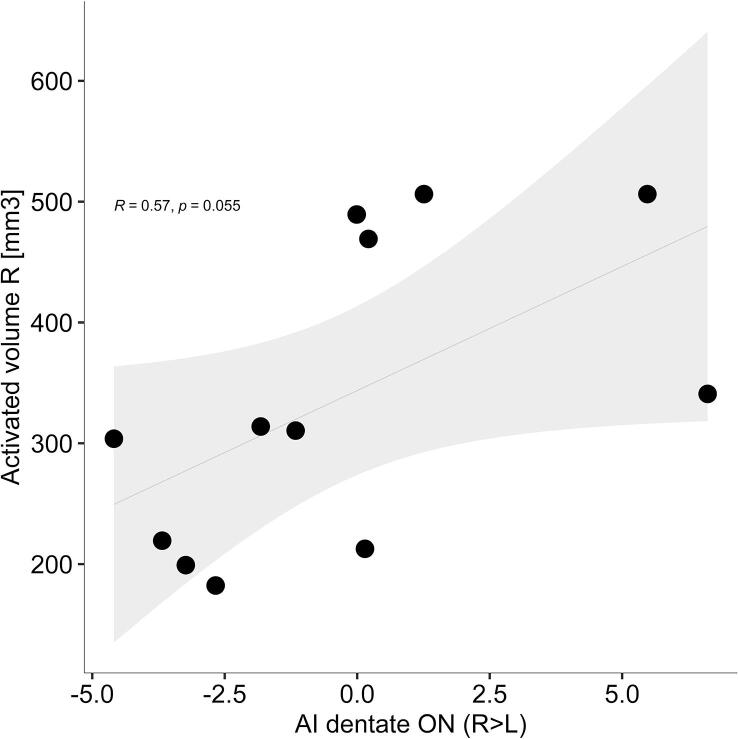
Association between asymmetry index of the dentate nuclei (AI-D) with volume of activated tissue (VAT) on the right side. Each dot represents individual data. Grey line and area represent the regression line and corresponding 95% confidence interval. r, Pearson correlation coefficient; p, significance value. Positive values of AI-D indicate more right-sided dentate activity and vice versa.

Metabolic activity in the defined ROI was compared between hemispheres by AI in twelve participants. AI of DN (AI-D) during DBS stimulation was trend level positively associated with the VAT size on the right side (r = 0.56, p = 0.055, Fig. 5) indicating that in patients with larger right VATs, glucose metabolism was also higher in the right compared to the left DN. This association became significant when participants’ age, sex, disease duration and time since implantation were taken into account (AI-D: t = 2.85, p = 0.029).

AI-D was not associated with DRTx/DRTu ratios, even when adjusted for other clinical parameters. AI of thalamus showed no relevant correlation to tractography-derived parameters.

## 4 Discussion

We report disbalanced recruitment in the right VAT favoring DRTu over DRTx being associated with DTE and ataxia in the later course (≫12 months) of thalamic DBS for ET. This is supported by a trend for pronounced metabolic activity in the right DN. Further exploratory findings denote that VAT coverage of right cerebellar outflow fibers in general might determine this phenomenon.

### 4.1 Efficacy of thalamic DBS in essential tremor

Exploratory analyses of ON_12M_ data suggest that these associations are important for long-term efficacy only and are less relevant in the early postoperative course. This is supported by the work of Tsuboi et al. (2021) who found coverage of DRTx and DRTu to be equally efficacious in the very early course (6.6±1.8 months (mean ± SD)) of thalamic DBS. Similarly, Deuter et al. (2022) found smaller distances to both DRT portions for effective contacts regarding postural tremor one month postoperatively.

Several studies denote that efficacy is linked to a more anterior stimulation site (albeit at trend level for Pilitsis et al.) (Germann et al., 2023, Hidding et al., 2019, Middlebrooks et al., 2018, Pilitsis et al., 2008, Sandoe et al., 2018), but among them, only Sandoe et al. (2018) focussed on long term outcomes (≫12 months). Although we did not observe an association in terms of MNI y coordinates in our cohort, the correlation with the ratio DRTx/DRTu complements earlier findings, as it points in the same direction with DRTx being situated anteriorly to DRTu and may thus serve as the common anatomical explanation.

The concept of DTI-based tractography of the DRT to explain therapeutic efficacy as proposed by Coenen et al., (2011b) has been substantiated by numerous studies (Akram et al., 2018, Al-Fatly et al., 2019, Anthofer et al., 2017, Brinda et al., 2023, Coenen et al., 2020, Coenen et al., 2014, Dembek et al., 2020, Deuter et al., 2022, Nowacki et al., 2022, Petry-Schmelzer et al., 2020). We now provide new insight into how proportional stimulation of DRTx and DRTu affects long-term postoperative outcomes which is not covered by the aforementioned studies. However, this is of paramount importance for targeting and DBS electrode implantation, since our and others’ previous results suggest that intraoperative testing is not feasible to differentiate DRTu and DRTx stimulation being equally effective at this (extremely early) timepoint. Moreover, commercially available planning softwares only allow for deterministic fiber tracking, which is unable to robustly detect the DRTx (Coenen et al., 2021, Schlaier et al., 2017).

Our exploratory analyses suggest a positive association between measures of stimulation intensity and DTE (Supplementary Fig. S1). Comparing patients with and without DTE, Pilitsis et al. (2008) found similar differences at trend level, while Sandoe et al. (2018) did not observe significant differences between the groups regarding stimulation voltage or pulse width. Both studies, however, differ methodologically from our study in their definition of DTE and design of analysis impeding a direct comparison. The close associations between measures of stimulation intensity and measures of DRT coverage (cf. Supplementary Fig. S1) did not allow us to further differentiate their role for DTE. Though, at the same time, the nature of their associations (negative and positive, respectively, cf. Supplementary Fig. S1) which is constantly found over time (cf. Supplementary Figs. S1 and S2) underline the relevance of electrode location resulting in the differential DRTx and DRTu coverage present in our sample. This raises the question of why this locational aspect is not reflected by according associations with MNI-y coordinates in our sample. We attribute this to the parabola-shaped course of the DRT in the sagittal plane with the typical stimulation site of VIM/cZI-DBS being situated at the turning point. As a result, the course of the DRT as a prior explains tremor improvement better than a single linear coordinate (Middlebrooks et al., 2021, Sammartino et al., 2016).

In our sample, DTE is linked to signs of ataxia. While these entities are sometimes segregated and examined differentially (Chiu et al., 2020), there certainly is an overlap (Chiu et al., 2020, Merchant et al., 2018). Based on our previous findings (Sajonz et al., 2022), we are confident that DTE is a process that eventually entails ataxia often starting with slightly changed tremor features (intentional, more proximal, lower frequency), that may be still subsumed to ET by others. We also observed a strong association of a low-frequency rebound tremor (after switching off stimulation) with DTE and ataxia (Sajonz et al., 2022), whereas Chiu et al. (2020) classified tolerance (as opposed to ataxia) by loss of efficacy and/or evidence of rebound.

The consideration of DTE and ataxia (but not ataxia due to sudden supratherapeutic stimulation (cf. Sajonz et al., 2022)) as aspects of the same process differing in stages across patients would reconcile the variation in clinical findings with regard to symptoms and timing of their recovery, too. Based on the data of our sample, we cannot differentiate whether stimulation intensity was the cause of DTE or whether stimulation intensity was increased in response to a tolerance development leading eventually to DTE (and ataxia).

### 4.2 Anatomical considerations on crossed and uncrossed dentato-rubro-thalamo-cortical projections

The cerebellum projects via the superior cerebellar peduncle to the contralateral thalamus and further to the motor and premotor cortices. The origin of these fibers are the deep cerebellar nuclei (DN, globose and emboliform nuclei) with DN being the main source (Nieuwenhuys et al., 2008). The lower presynaptic part of this fiber pathway is called the fasciculus cerebello-thalamicus (Gallay et al., 2008). The postsynaptic part is a thalamo-cortical projection (Kievit and Kuypers, 1977, Nieuwenhuys et al., 2008). If the post-synaptic extension of this fiber-pathway to the cortex is jointly regarded, the term DRT (dentato-rubro-thalamo-cortical tract) has found entry in the literature. The DRT represents a distinct part of the tremor network and is a rather neurosurgical driven structural description (Coenen et al., 2011b). Crossing of the fibers to the contralateral hemisphere occurs at the level of the commissure of Wernekinck (Voogd and van Baarsen, 2014) below the nucleus ruber level (Fig. 3D) and thus in the presynaptic fasciculus cerebello-thalamicus. In a cadaveric microdissection study a proportion of 20 % of the superior cerebellar peduncle fibers were located ipsilateral, indicating a dominant proportion of crossing fibers (Meola et al., 2016). It has been established with retrograde injection studies in the cat, that crossed and uncrossed projections are collateral axons *of the same DN neuron* (Ilinsky et al., 1987). Assuming that this anatomy applies to humans, then antidromic propagation effects – as established by Grill et al. (2008) in a computational model – could explain how disbalanced stimulation favoring DRTu over DRTx in the non-dominant thalamus potentially causes a stimulation overload on the dominant rather than the non-dominant DN outflow, while at the same time causing more problems in the non-dominant hand. This concept is further strengthened by evidence of cerebellar asymmetry with non-dominant hand movements requiring bilateral cerebellar involvement (Hu et al., 2008).

The additional uncrossed portion of 20 % of the fibers within the DRT (Meola et al., 2016, Petersen et al., 2018) is a rather recent concept which finds some verification in the human both anatomically and electrophysiologically (Brogna et al., 2022, Meola et al., 2016). It has to be pointed out, though, that at this moment the evidence for the existence of DRTu in humans has to be regarded as circumstantial, coming from DTI studies, microdissection in human postmortem specimens and stimulation experiments. In imaging studies the differential parts of the projection appear to reach distinct regions of the ventral thalamus. Petersen et al. (2018) used DWI and functional magnetic resonance imaging (fMRI) techniques and revealed a bias of connectivity with more medial and posterior thalamic projections for DRTu (VPM, VPL, CM – in Morel’s nomenclature (Krauth et al., 2010) – corresponding to Hassler’s somatosensory/vestibular territory Vca and CM nuclei) and more anterior and lateral for DRTx (VM, VPi, VLA, corresponding to Hassler’s Vop, Vim and Voi nuclei (Ilinsky and Kultas-Ilinsky, 2002)). Even if not fully proven, the DRTx/DRTu dichotomy in function and extension of their projection to thalamus and cortex therewith offers support for our findings.

### 4.3 Evidence from lesion surgery and lesion studies

**Fig. 6:**
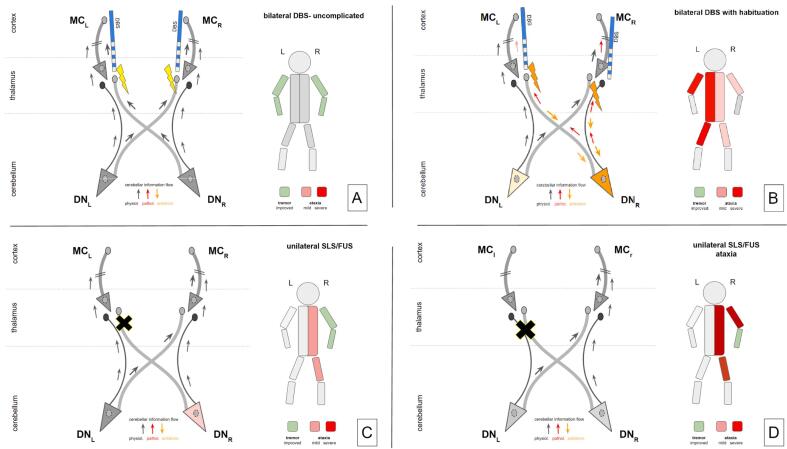
A-D. Different scenarios for the development of ataxia through cerebellar involvement under deep brain stimulation (DBS) or stereotactic lesion surgery (SLS) differentially addressing the crossed and uncrossed portion of the dentato-rubro-thalamic tract (DRT). A, Uncomplicated and bilateral DBS. High frequency stimulation occurs at both crossed portions of the DRT. Stimulation energy is moderate. As such, the system is permissive for the physiological cerebellar signal while dampening tremor signals. As a result, tremor is improved. B, In this scenario, the right-sided DBS electrode is positioned slightly medial and posterior (not shown). Tremor re-emerged over time and therapeutic stimulation energy was increased (orange lightning). Antidromic interference via DRTx (left thalamus) and DRTu (right thalamus) with the right dentate neuronal population leads to pathological information flow into bilateral thalami (left > right). As a result, ataxia emerges together with more cerebellar mixed tremor especially on the right side and loss of tremor control due to less efficacy of DRTu for the intention tremor component. Both dentate nuclei will be involved in these mechanisms leading to potentially more left sided ataxia because of the bilateral involvement of the cerebellum in the non-dominant (body) side. Of note: increase of right thalamic stimulation for control of tremor in the non-dominant upper extremity can – via this mechanism – also lead to increased limb ataxia on the dominant upper extremity. C, Optimally located lesion addressing the left thalamus (DRTx) only. Physiological information flow from the right dentate is perturbed but (partially) recovered via the left dentate (DRTu). Good tremor response is the result (potentially with some mild right-sided ataxia). D, Left thalamic lesion encroaches on DRTx & DRTu. Physiologic information flow to the left motor cortex (MC) is disturbed leading to ataxia with some good tremor response. Legend: DN, dentate nucleus; MC, motor cortex; DBS, deep brain stimulation; SLS, stereotactic lesion surgery; FUS, focussed ultrasound. (For interpretation of the references to color in this figure legend, the reader is referred to the web version of this article.)

A recent review of the focused ultrasound literature reports a 30 % rate of cerebellar ataxia potentially related to posteriorly located lesioning encroaching on the DRT (Agrawal et al., 2021). DWI application in focused ultrasound lesioning ataxia side effect workup remains unclear with respect to differential DRTx/DRTu involvement (Boutet et al., 2018), since only DRTx is regarded. However, cumulated overlap lesion volume with DRTx allows the conclusion of additional DRTu lesioning (in posterior proximity to DRTx, Fig. 4c in (Boutet et al., 2018)) being causative. We are aware, however, that DBS and lesioning exert their treatment effects based on different physiological mechanisms (cf. Fig. 6). So lesion and DBS/VAT locations might be not simply comparable. German et al. come to similar conclusions (Germann et al., 2023).

Unilateral lesions of the entire cross-section of the DRT typically lead to unilateral ataxia (Marek et al., 2015) mimicking cerebellar lesions. Unilateral cerebellar lesions are known to typically cause only ipsilateral limb ataxia. Comparing patients after unilateral cerebellar stroke and normal controls, Immisch et al. (2003) found impairment in limb movements both ipsilateral and contralateral to the lesion. The contralateral side was involved to a lesser extent, though. As a possible confirmation, in a single case with recovery of right-sided ataxia (after right sided cerebellar damage) through DBS of the contralateral intact DN, Teixeira et al. (2015) demonstrated a result that could be explained through symptom recovery via modulation of the supposed ipsilateral uncrossed DRT to left thalamus and motor cortex. As a consequence for our results here, bilateral thalamic suprathreshold DBS in ET might lead to the disturbance of physiological cerebellar signal flow to both hemispheres and therefore might serve as unilateral or bilateral functional cerebellar lesions leading to ataxia (Fig. 6). With respect to handedness, the information on cerebellar hard-wiring is sparse (Hu et al., 2008). Non-dominant movements need the surveillance and involvement of both cerebellar hemispheres. Most consistently, Hu et al. (2008) note in their review that movements of the dominant hand are realized via involvement of the ipsilateral cerebellar hemisphere (in all our cases the right cerebellum). In line with this, our findings point exactly toward this special role for the right (=dominant) cerebellum.

### 4.4 Proposed mechanism for delayed therapy escape

Based on our results and recent other studies, we conclude that DRTu seems to be a Janus-faced structure offering tremor improvement initially, whereas in the long run its stimulation is not efficient for intentional tremor and may even elicit features of ataxia, among them intentional tremor. In the setting of a right handed patient with stimulation favoring DRTx over DRTu in the dominant thalamus and vice versa we propose the following course as potential mechanism evolving gradually over years:

1. With deteriorated tremor over time (e.g. due to habituation, disease progression, etc.) stimulation is increased in the left thalamus to maintain tremor control for the right hand, which is usually more in focus of attention in right handed patients. This entails predominantly DRTx coverage improving both postural and intentional tremor and only moderate additional DRTu coverage affecting outflow of the left DN, but reaches a supratherapeutic overload together with DRTx coverage in the right thalamus and thus leads to subtle ataxic features including intentional tremor on the left side. This may be aggravated by the need for bilateral cerebellar involvement in non-dominant movements.
2. Subsequently, right thalamic stimulation is increased, but due to a vast amount of DRTu covered this does not sufficiently control the intentional tremor component on the left. Moreover, increased DRTu coverage results in a supratherapeutic overload in the right DN and ataxic features including increased intentional tremor on the right side.
3. The process becomes a vicious circle jumping to step 1 again. In addition, neuroplastic changes are involved producing rebound tremor rather than improvement after switching stimulation off.

### 4.5 Limitations

The inclusion of retrospective data, which was necessary to determine therapy efficacy and DTE, respectively, entails missing values, so some analyses could be performed in subgroups only and have to be interpreted with care.

In participants with in-patient stimulation adjustment involving new introduction of pulse width reduction, we included only retrospective data acquired just before these changes instead of the prospective data acquired at the study appointment. By this measure, however, bias can be reduced arising from the two modes of action for pulse width reduction (VAT size (Butson et al., 2007) vs. chronaxie-based fiber selection (Groppa et al., 2014)) the interrelation of which remains to be confirmed (cf. Ng et al., 2023).

We are aware that DTE has also been linked to several further risk factors, but it is beyond the scope of this study to further differentiate their impact in addition to DRT coverage.

This study did not employ neuropsychological inventories or fMRI paradigms to determine hand dominance or hemispheric dominance, which would have been more precise than simply asking the patient. As a result, we cannot exclude that right-hemispheric dominance is present in some of the analyzed patients. However, we anticipate that this would rather dilute the power of our model.

## 5 Conclusions

DTE after thalamic DBS for ET is a rather common phenomenon and poses significant burden on patients and care-givers. We here present evidence that part of the symptom complex might be related to the mere DBS electrode position and consecutive stimulation. Depending on the hemisphere, DRTx and DRTu can be addressed in a disbalanced fashion leading to DTE. In our interpretation, a pronounced involvement of the DRTu fibers in the right VAT leads to a vicious circle of inferior efficacy for intentional tremor on the left in the long run combined with an antidromic overactivation of the right cerebellum with a consecutive and stimulation induced cerebellar syndrome. In this respect, it is the role for the right-sided cerebellum in its bilateral function that dictates ataxia evolution over time. Thus, targeting electrode positions that prevent the overstimulation of DRTu fibers offers a potential solution to DTE, especially in bilateral implantations. The role of the differential involvement of DRTu/x components is the focus of a prospective clinical trial (DRKS00032400).

## CRediT authorship contribution statement

**Bastian E.A. Sajonz:** Conceptualization, Methodology, Validation, Formal analysis, Investigation, Resources, Data curation, Writing – original draft, Visualization, Project administration. **Marvin L. Frommer:** Software, Investigation, Writing – review & editing. **Marco Reisert:** Writing – review & editing, Visualization, Software. **Ganna Blazhenets:** Writing – original draft, Visualization, Validation, Software, Methodology, Investigation, Formal analysis, Data curation. **Nils Schröter:** Writing – review & editing. **Alexander Rau:** Writing – review & editing. **Thomas Prokop:** Writing – review & editing. **Peter C. Reinacher:** Writing – review & editing. **Michel Rijntjes:** Writing – review & editing. **Horst Urbach:** Writing – review & editing, Resources. **Philipp T. Meyer:** Supervision, Resources, Writing – review & editing. **Volker A. Coenen:** Writing – original draft, Visualization, Supervision, Methodology, Conceptualization.

## Declaration of competing interest

The authors declare that they have no known competing financial interests or personal relationships that could have appeared to influence the work reported in this paper.

## Data Availability

Data will be made available on request.
